# Spatial behavior of socially isolated wild pigs ( *Sus* scrofa) following sounder removal via trapping

**DOI:** 10.1002/ps.70630

**Published:** 2026-03-12

**Authors:** Sebastian Gomez‐Maldonado, Matthew T McDonough, Jonathon J Valente, Mark D Smith, Stephen S. Ditchkoff

**Affiliations:** ^1^ College of Forestry, Wildlife and Environment Auburn University Auburn AL USA; ^2^ U.S. Geological Survey, Alabama Cooperative Fish & Wildlife Research Unit, College of Forestry, Wildlife and Environment, Auburn University Auburn AL USA

**Keywords:** pest management, disease management, integrated pest management, movement ecology, spatial behavior, wild pig management

## Abstract

**BACKGROUND:**

The rapid expansion of wild pig (*Sus scrofa*) populations across North America, coupled with increased concern over disease transmission, has intensified the need for effective management strategies. Trapping is widely regarded as the most effective control method; however, trapping events often fail to capture entire sounders. The behavioral responses of untrapped individuals following partial sounder removal remain poorly understood, potentially undermining management efficiency. We evaluated the spatiotemporal movement responses of wild pigs that became socially isolated following trapping events.

**RESULTS:**

We deployed GPS collars on 18 female wild pigs from multiple sounders and quantified post‐trapping movement patterns using distance from trap site, step length, persistence velocity, space covered and overlap area over a 30‐day period. Movement responses were highly variable among individuals, but wild pigs travelled an average of 1.2 km from the trap, with a maximum observed distance of 6.37 km. Space‐use overlap was limited, and range sizes remained relatively stable. Individuals originating from sounders with a greater proportion of females moved farther from the trap, whereas wild pigs in better body condition exhibited lower movement velocities.

**CONCLUSION:**

Socially isolated wild pigs generally remained close to trap sites following partial sounder removal and rarely dispersed from the area. This behavioral pattern suggests a predictable post‐trapping window during which untrapped individuals remain spatially accessible. These findings provide critical empirical support for adaptive trapping strategies, indicating that follow‐up removal efforts can be effectively concentrated near original trap locations to improve management efficiency and reduce the risk of population persistence or disease spread. © 2026 The Author(s). *Pest Management Science* published by John Wiley & Sons Ltd on behalf of Society of Chemical Industry. This article has been contributed to by U.S. Government employees and their work is in the public domain in the USA.

## Graphical abstract

Following partial sounder removal, socially isolated wild pigs maintained site fidelity near traps, highlighting post‐control behavioral tendencies that are relevant to pest management and disease mitigation.

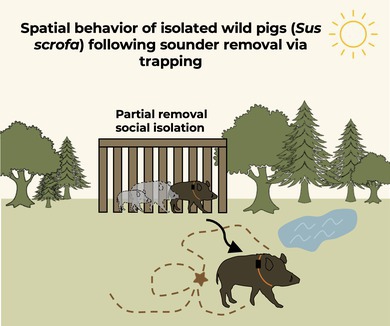

## 1 INTRODUCTION

The wild pig (*Sus scrofa*), a successful invasive species in North America and other continents, has garnered significant attention in the United States in recent decades owing to the accelerated rate at which it has expanded its range. As a result, substantial effort has been directed at developing management practices and tools to decrease environmental impacts,^1^, ^2^, ^3^ reduce population densities,^4^ and mitigate the spread of diseases that could impact livestock, wildlife and humans.^5^, ^6^ The potential for wild pigs to spread foreign animal diseases has always been a concern, but it has increased recently owing to the rapid spread of African swine fever (ASF) in wild swine populations in Europe and other regions, as pigs serve as both reservoir hosts and highly mobile vectors for this environmentally persistent virus.^7^, ^8^

Although management of wild pigs involves a variety of tools and techniques to reduce their impacts, most efforts are focused on density reduction, and trapping is the primary form of population control employed across most of their range.^9^ Most successful trapping programs incorporate some form of removal strategy,^10^ with whole sounder removal being the strategy that has garnered the most attention. Recent studies have demonstrated that whole sounder removal can significantly reduce wild pig numbers, especially in areas where wild pigs exhibit high site fidelity.^11^, ^12^, ^13^ However, the success of such programs in North America have been inconsistent, perhaps indicating that sustained and long‐term efforts are essential for reducing populations.^14^ Additionally, the spatial and social dynamics of wild pigs complicate the effectiveness of whole sounder removal. Although sounders are generally cohesive, individual pigs may exhibit behaviors such as temporarily splitting from the group, shifting home ranges or overlapping with other sounders.^15^ These patterns increase the likelihood of partial removals, specifically in areas with complex landscapes or dense cover, where pigs can evade traps. As a result, even targeted efforts may leave behind untrapped individuals, who can rapidly recolonize and compromise control programs.^11^

When implementing whole sounder removal strategies to manage wild pig populations, there is potential disruption to sounders. For instance, trapping may not capture all individuals within the sounder, despite efforts to do so, leading to possible compensatory responses by untrapped individuals. Limited research suggests that when some members of a sounder are trapped and removed, the behavior of the remaining, untrapped individuals may shift in response. Such social disruption can lead to changes in home range size^11^ or a tendency for pigs to remain closer together, thereby reducing group dispersion.^16^ Further, recent research suggests that social interactions are more important than land‐cover variables to movement behavior of wild pigs^17^; thus, effects of trapping on movement behavior could be impacted by social structure. Given the role social structure poses on wild pig behavior, understanding social disruption is crucial for improving trapping strategies, particularly during disease emergencies such as the introduction of ASF.

Our goal was to evaluate the effect of trapping on spatiotemporal behavior of wild pigs. Specifically, we focused on the behavioral responses of individual wild pigs that remained after the rest of their sounder had been euthanized during trapping. We examined key movement metrics—such as distance traveled from the trap, step length, persistence velocity, space covered, and overlap proportion of area after the trapping events—to determine whether they changed over time following the trapping event. Understanding temporal variation in these metrics is important because wild pigs may exhibit immediate behavioral responses to social disruption that fluctuate over time. These dynamics can influence the timing and effectiveness of follow‐up management efforts, especially during disease emergencies when the risk of spread may vary depending on when pigs resume routine movements. By quantifying these patterns, we aim to improve predictions of post‐trapping space use and assess how behavioral recovery may affect the success of control programs targeting disease outbreaks.

## 2 MATERIALS AND METHODS

### 2.1 Study area

**Figure 1:**
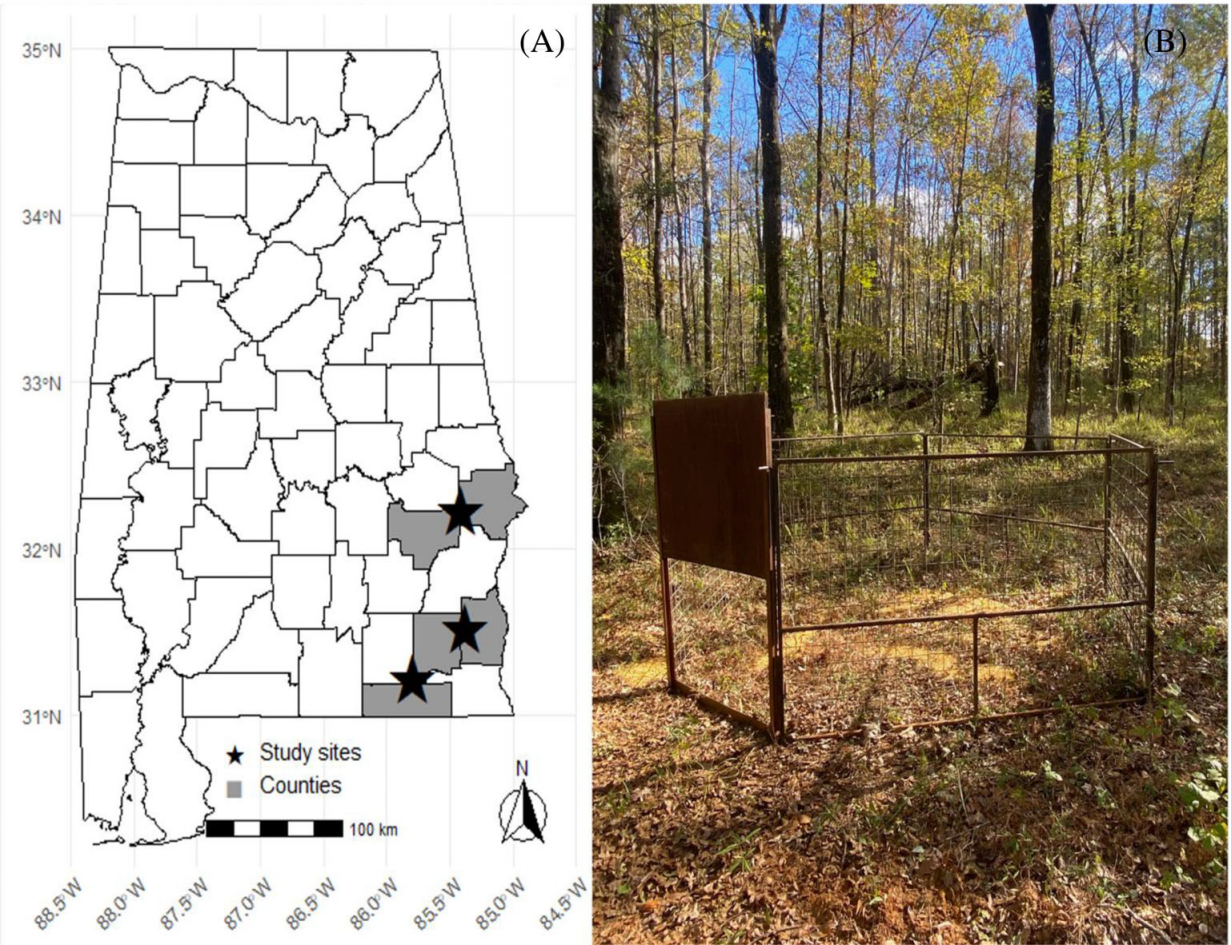
Spatial layout of wild pig (*S. scrofa*) study sites and trap set up. (A) Map of Alabama showing five focal counties (Bullock, Russell, Dale, Henry and Geneva) used for GPS‐collar deployment on wild pigs between 2022 and 2023. Shaded areas indicate counties where wild pigs were GPS‐collared following sounder removal events. Black stars mark approximate capture sites. (B) Field photograph of the corral‐style trap used for live captures of wild pigs, illustrating the guillotine‐style door, panel configuration, and surrounding habitat.

The research took place on three study sites encompassing five counties (Bullock, Dale, Geneva, Henry, and Russell) in Alabama, USA [Fig. 1(A)]. All properties within the study areas were selected owing to their participation in the USDA Natural Resources Conservation Service's Feral Swine Eradication and Control Pilot Program^18^ and/or because they were cooperators with USDA Wildlife Services. All study sites were in the coastal plain region of Alabama, where the landscape consisted of a mosaic of agricultural fields, mixed forest, and wetlands. Typical agricultural activities included row‐crop production (e.g. corn, peanuts, soybeans, cotton) and cattle operations. Vegetation in these regions was primarily composed of dominant overstory species such as loblolly (*Pinus taeda*), slash (*P. elliottii*), longleaf (*P. palustris*) and shortleaf pine (*P. echinata*), interspersed with occasional patches of mixed hardwoods, whereas understory composition varied locally. Elevation among study areas ranged from 104 to 201 m; average temperature was 19.2 °C (range 11.7–26.2 °C) and annual precipitation was 2.7 m.^19^

### 2.2 Data collection

From 22 February 2022 to 1 March 2023, we conducted opportunistic wild pig captures using corral traps baited with whole kernel corn [Fig. 1(B)] with the aim of capturing different sounders throughout the study sites. Once sounders were located, corral traps were assembled on site and consisted of five panels (each 4.9‐m long) secured with 1.6‐m t‐posts firmly anchored into the ground on the exterior. Panels were positioned with a 0.5‐m overlap and affixed to t‐posts using baling wire. Two types of trap doors were employed. At most sites, we used a 2.4‐m guillotine‐style gate (M.I.N.E.™ Gate; Jager Pro, Fortson, GA, USA) triggered remotely by an observer monitoring live footage from a camera system (M.I.N.E.™ Cam; Jager Pro) between 17:00 and 06:00 h. In other locations, we deployed traditional trip‐wire triggered guillotine doors. These traps used a tensioned wire suspended 40–50 cm above ground level across the rear section of the trap, designed to be triggered by adult wild pigs exerting pressure on the line. This design minimized premature activation by smaller pigs or nontarget species. Wild pigs were allowed to acclimate to the trap until the entire sounder reliably entered the trap. Once the entire sounder was consistently inside the trap, the gate was either remotely closed by an observer or, in the case of the trip‐wire guillotine design, set the previous evening to allow wild pigs to trigger it themselves.

After capture of the sounder, we randomly selected one female adult wild pig (*c*. 40 kg) and affixed it with a satellite transmitting collar (Telonics® GPS/Iridium Iridium collars TGW‐4470‐4; Telonics Inc., Meza, AZ, USA). We focused only on females in this experiment because they are generally the target for wild pig control programs.^20^ To attach the collar, we isolated the selected wild pig from the rest of the sounder then blindfolded and immobilized it using lassos to reduce stress without the use of sedatives or anesthesia. We collared only one individual wild pig per sounder. We programmed the GPS collars to collect and store locations every 15 min for monitoring. Drop‐off mechanisms were programmed to automatically release the collars 180 days after deployment, after which we retrieved the collars and downloaded the full location datasets. After collaring, we released the selected pig from the corral trap and euthanized the remaining wild pigs within the sounder. We recorded sounder size and composition (males/females; adults/juveniles) and measured chest girth and body length of both the collared wild pigs and other sounder members (following Kruis *et al*.^21^).

We carried out trapping efforts with the assistance of U.S Department of Agriculture Animal and Plant Health Inspection Service (USDA APHIS) Wildlife Services collaborators. No additional trapping occurred at or immediately adjacent to the initial trapping/release sites during the study period. Before data collection, collaborators requested that landowners and hunters refrain from targeting our selected wild pigs. However, landowners and hunters were permitted to remove other wild pigs at their discretion as part of normal operations. None of the study areas had been trapped previously by Wildlife Services, but wild pigs in these areas faced pressure from sporadic trapping, shooting, and hunting with dogs. We conducted all capture and handling procedures under approval from the Institutional Animal Care and Use Committee (IACUC) at Auburn University under folio PRN 2022–4026.

### 2.3 Spatial movement metrics

We cleaned spatial data from GPS‐collared wild pigs by removing erroneous fixed locations and duplicates. We established the final recorded locations as either the first mortality signal received from the collar (if deceased) or the programmed release time of the collar. For each individual, we examined daily variability in five spatial movement metrics following release from the trap: (i) distance from the trap, (ii) step length, (iii) persistence velocity, (iv) space covered and (v) overlap area. We selected these spatial metrics based on their demonstrated utility as behavioral indicators—previously used in other taxa—that could be displayed by wild pigs following a trapping event under the context of its movement ecology.^22^ Each metric captures a distinct behavioral dimension. Distance from the trap reflects attraction or aversion to the site of capture and may indicate immediate avoidance behavior (e.g.,^23^). Step length is used as an indicator to assess the spatial displacement of individuals, revealing changes in movement efficiency or caution (e.g.^24^). Persistence velocity—scaled to m per day—integrates both speed and directional consistency to capture the magnitude and tendency of movement to persist in a given direction, thereby distinguishing between straight, goal‐oriented movements and short, localized movements (e.g.^25^, ^26^). Space covered represents the daily extent of area used and can signal exploratory movements or range contractions (e.g.^27^). Overlap of area evaluates day‐to‐day similarity in space use; greater overlap suggests stability and site fidelity,^28^ whereas reduced overlap may indicate behavioral displacement.^29^ Together, these metrics offer complementary insights into how wild pigs adjust their movement patterns following partial sounder removal. All analyses were conducted in R v4.3.2.^30^

We calculated distance from the trap as the straight‐line distance for each GPS point recorded and the trap location where each wild pig was captured. Step length was calculated as the linear distance between consecutive GPS points, representing individual displacement rates. Persistence velocity (*V*_p_) was calculated using the following equation: $V_{p}\left(T_{i}\right)=V\left(T_{i}\right)\cos\left(\theta \left(T_{i}\right)\right)$, where *T*_*i*_ represents each time observation, *V(T*_*i*_) represents the scalar speed (i.e. magnitude of the velocity vector) at time *T*_*i*_, and θ(*T*_*i*_) is the angle between the velocity and a reference direction at that time. Greater values represent more directed and efficient movement patterns—such as dispersal or relocation—whereas lesser values indicate more frequent directional changes, typically reflecting foraging or localized searching movement patterns.^25^, ^31^

We calculated space covered as the total ground area used by wild pigs at a daily scale following release from the trap, using autocorrelated kernel density estimation (AKDE) via the ctmm package.^32^ The AKDE accounts for the spatial and temporal autocorrelation in movement data (i.e. repeated observations of the same pig) when developing an estimate of the space used by that pig.^33^ We selected initial autocorrelation parameters from the variograms of each individual wild pig using the *guess()* function, then used the *ctmm.select()* function to fit suitable models via maximum likelihood. We compared those models using Akaike's information criterion (AIC) values (Supporting Information Appendix S1) and used the most parsimonious model to estimate the total ground area used by the pig with a 95% utilization distribution (UD). We calculated the overlap proportion across six fixed time periods (days 1, 5, 10, 15, 20 and 30) using 15 pairwise comparisons (e.g. day 1 *versus* day 5, day 1 *versus* day 10, etc.). We computed 95% UDs using the adehabitat package^34^ and used the *kerneloverlaphr()* function with the ‘HR’ index^35^ to quantify spatial overlap. Because our objective was to quantify short‐term space use following isolation rather than to estimate full home ranges, we derived daily utilization distributions for each pig based on 95 high‐frequency locations collected over each 24‐h period. This sampling density is sufficient to characterize space use over that specific day, even though it is not intended to represent a full home range. We therefore treated these as daily space‐use polygons, not home‐range estimates, and used them only to calculate the proportion of spatial overlap between selected post‐trapping days.

### 2.4 Short‐term temporal variation analysis

We used generalized linear mixed‐effects models (GLMM; nlme package^36^;) to examine changes over time in four of the spatial parameters (distance to trap, step length, persistence velocity, and space covered) over specific time points: days 1, 5, 10, 15, 20, and 30 post‐trapping. Our GLMMs were built using one of the spatial parameters as the response variable and time points (treated as continuous variable) as fixed effects. Pig ID was included as a random effect in all models to account for individual variability in these spatial parameters. Because these time points were fixed and not collected as a daily time series, this analysis differs structurally from the continuous long‐term models described below.

We assumed a normal distribution for distance from the trap, persistence velocity and space covered, and a negative binomial distribution for step length (Appendix S2). To identify the appropriate statistical distribution, we compared models that assumed normal, Poisson and negative binomials for each response variable. We evaluated the fit of each model by comparing a *χ* ^2^ test statistic from the original dataset to a distribution of *χ* ^2^ statistics derived from 500 datasets generated via parametric bootstrapping from the fitted models. A model was considered to be a reasonable fit if χ^2^ did not fall within the upper or lower 0.025 quantiles of the simulated *χ* ^2^ distribution.^37^ This procedure was repeated to identify appropriate statistical distributions for every response variable.

We additionally performed a Kruskal–Wallis^38^ test on each response variable to determine if there was a specific time frame where wild pigs drastically changed their spatial behavior after the trapping event. The Kruskal–Wallis test is a nonparametric analysis of variance that evaluates differences based on ranks of the data to determine whether there are statistically significant differences between the six time points, assuming all wild pigs behave likewise. We chose this nonparametric test because not all pigs had data for all time points, resulting in low sample sizes for some comparisons.

We also used a Kruskal–Wallis test^38^ to assess whether the proportion of area overlap differed significantly among the 15 combinations of time points described above. To further explore temporal patterns in spatial consistency, we implemented a logistic regression model to estimate the probability of overlap exceeding a specified threshold (>30%) at all time points. This threshold was selected based on previous studies of wild pig territoriality^39^ and space‐use overlap,^40^ which identified 30% as a meaningful level of shared space use. Overlap values above this threshold have been interpreted as indicative of spatial fidelity or reuse, whereas lower values may reflect avoidance or shifting home ranges. Because spatial overlap is a pairwise, noncontinuous measure that was calculated across the specific time‐point combinations rather than daily, it was analyzed separately from the four continuous daily movement metrics. We created a binary response variable for each pairwise comparison (1 = overlap ≥0.3; 0 = overlap <0.3). The logistic regression model was built using the binary variable as the response variable, with Time 1 (six levels) and Time 2 (six levels) included as categorical fixed effects representing the two time points in each pairwise comparision and their interaction.

### 2.5 Social structure and long‐term temporal variation analyses

Given that spatial overlap was modeled separately owing to its pairwise and threshold‐based structure, we restricted our long‐term temporal variation and further analyses to the four continuous, daily resolved spatial parameters: distance to trap, step length, persistence velocity and space covered. We used GLMMs (nlme package^36^) to examine the influence of social structure (i.e. sounder size and composition) on the four continuous spatial parameters over extended periods following the trapping event. To complement our short‐term analysis of the first 30 days post‐trapping, we included the number of days since the trapping event as a continuous covariate to evaluate longer‐term temporal variation. This allowed us to assess whether spatial parameters exhibited consistent increases or decreases over time across the entire post‐trapping period (ranging from 7 to 138 days), as pigs recovered or re‐established routine movement. Pig ID was included as a random effect to account for individual variability and to address pseudoreplication arising from repeated daily observations. Appropriate statistical distributions for each response variable were determined using the method described above (Appendix S2). Given that we were working with time series data (i.e. data collected subsequently over time such as real‐time tracking) we examined the residuals of our fitted models for temporal autocorrelation using the *acf()* function from the stats package.^30^ Mixed models with temporal autocorrelations were updated by adding an autoregressive process of order 1 (corAR1) as a correlation structure argument,^41^ and we evaluated model improvement by comparing models with and without the autoregressive residual process using the mumin package^42^ (Appendixes S4 and S5). Although some autocorrelation persisted across multiple lags (see Appendix S5), corAR1 structures are the standard option for mixed‐effects models in nlme and provide a reliable approximation of higher‐order temporal dependance in animal tracking data.^43^ Sounder size was defined as the total number of wild pigs in the sounder, excluding the collared wild pig. Sounder composition variables were the proportion of females and the proportion of adults in the sounder. We built GLMMs using one of the spatial parameters as response variables, and days after the trapping event (continuous), study sites (three levels), sounder size (continuous), sounder composition (continuous), and the interaction between the proportion of females and adults as fixed variables. Interactions were removed when not statistically significant. We assessed the significance of predictor variables in each GLMM using a type II ANOVA, which adjusts for the presence of multifactor models with interactions.^44^ We used GLMMs to assess whether individual body condition of each collared wild pig influenced post‐trapping behavior; we incorporated a body‐condition index into the GLMM framework. Fourteen pigs (78%) had complete body measurements. Because longer pigs are likely to have larger chests, we calculated a body condition index as the residuals from a regression of chest girth on body length.^45^ Positive values indicate better‐than‐average condition and negative values indicate poorer condition for a given body size. Because the limited sample size produced unbalanced groups, we retained body condition as a continuous predictor rather than categorizing pigs into ‘poor,’ ‘average’ or ‘good,’ as such grouping prevented model convergence. Body condition was added as an additional fixed effect to the social‐structure model, and model distributions and temporal autocorrelation structures were specified as described above (Appendixes S3 and S4).

## 3 RESULTS

We collared a total of 18 wild pigs from all study sites, with a mean tracking duration of 60.6 ± 10.4 days (mean ± SE), ranging from 7 to 138 days. Wild pigs had a mean of 4678.6 ± 800 fixed points, with a range of 462–84 215 points. The daily mean was 77.3 ± 3.7 GPS points, ranging from 25 to 90 points per day. Wild pigs were separated from sounders ranging in size from 2 to 18 (8 ± 1), and sounders comprised 58.2 ± 6.1% females (0–100; min–max) and 38.3 ± 9.2% adults (0–100%) (Table 1).

**Table 1:** Attribute data for each individual wild pig (*S. scrofa*) that was captured and fitted with a GPS collar in southeastern Alabama, USA, 2022–2023

Individual	Sounder size^†^	Sex proportion (F:M)^‡^	Age proportion (A:J)^§^	Body condition index	Start GPS	End GPS	Time monitored^¶^	No of fixed points
Pig 1	7	6:1	4:3	NA	22 February 2022	1 March 2022	7	634
Pig 2	7	5:2	2:5	−15.44	23 February 2022	26 March 2022	31	2552
Pig 3	2	2:0	1:1	−15.35	24 February 2022	29 April 2022	64	4403
Pig 4	17	12:5	2:15	−0.05	26 February 2022	5 June 2022	98	8523
Pig 5	10	6:4	3:7	NA	18 March 2022	22 June 2022	84	7563
Pig 6	18	11:7	1:17	5.38	1 April 2022	22 July 2022	118	7961
Pig 7	4	1:3	4:0	NA	9 September 2022	3 December 2022	85	7324
Pig 8	6	3:3	6:0	13.41	3 November 2022	2 March 2023	119	9361
Pig 9	6	5:1	2:4	NA	28 June 2022	22 July 2022	24	2046
Pig 10	9	6:3	3:6	−16.36	30 June 2022	7 July 2022	7	600
Pig 11	9	6:3	1:8	−12.02	19 July 2022	9 August 2022	21	1310
Pig 12	11	5:6	1:10	−5.61	27 September 2022	12 February 2023	138	9698
Pig 13	7	2:5	5:2	−4.19	1 October 2022	6 January 2023	97	6845
Pig 14	4	4:0	2:2	11.38	18 October 2022	23 January 2023	97	7714
Pig 15	8	4:3	8:0	−2.03	19 October 2022	26 October 2022	7	612
Pig 16	9	5:3	4:4	15.83	26 January 2023	3 March 2023	36	3254
Pig 17	6	3:2	0:5	3.43	26 January 2023	7 March 2023	40	3353
Pig 18	4	1:2	4:0	21.62	28 January 2023	15 February 2023	18	462

^†^ Sounder size at the time of capture, representing the total number of pigs in the sounder, with the collared wild pig included.

^‡^ Proportion of females (F) and males (M) within the sounder to which the collared pig belonged.

^§^ Proportion of adults (A) and juveniles (J) within the sounder to which the collared pig belonged.

^¶^ Total number of days the collared wild pig was monitored.

### 3.1 Short‐term temporal variation

**Figure 2:**
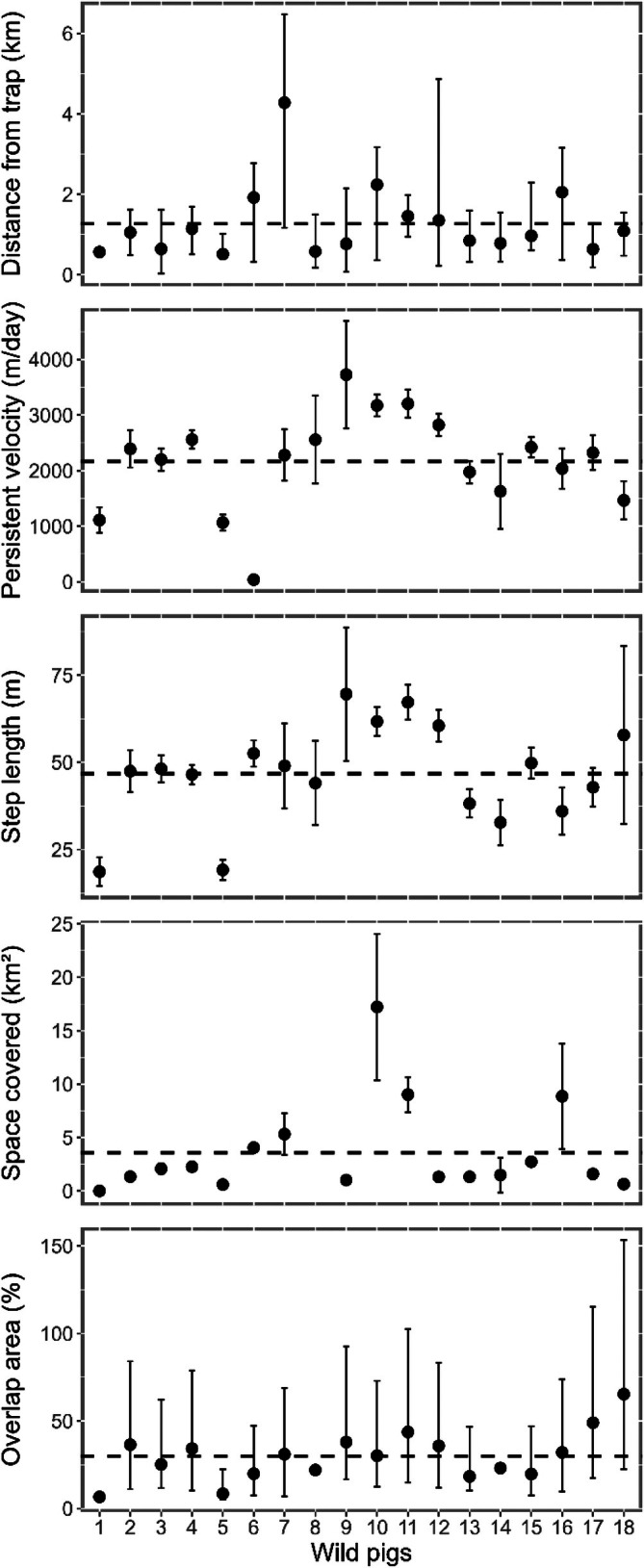
Mean (±95% CI) values of five spatial parameter from each individual wild pig (*S. scrofa*) collared across their monitoring period. Dashed line indicates the overall population mean.

**Figure 3:**
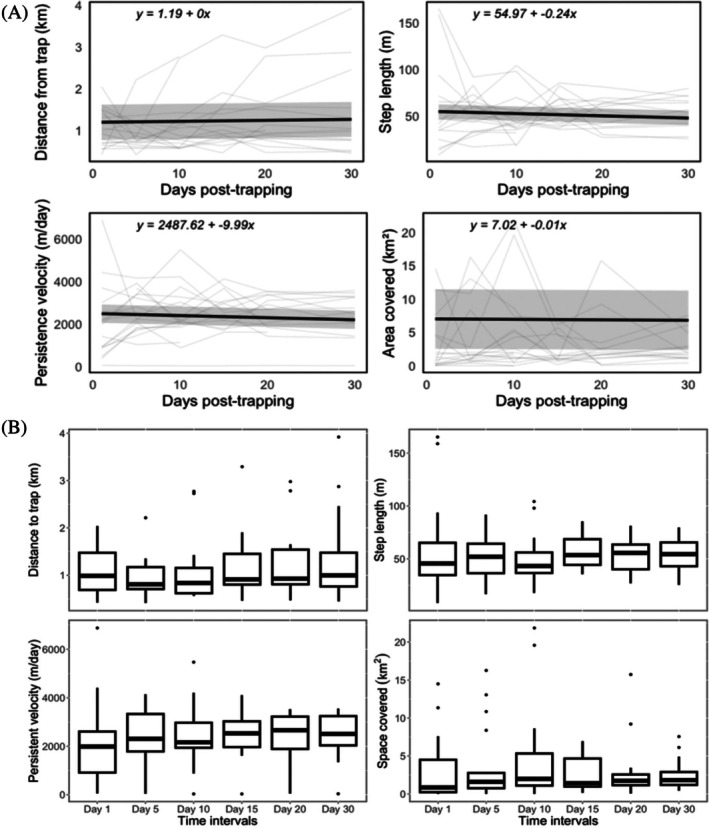
Temporal variation in spatial behavior of GPS‐collared wild pigs (*S. scrofa*) during the first 30 days following sounder removal in southeastern Alabama, USA (2022–2023). (A) Predicted temporal trends in distance from the trap site based on GLMMs. The solid black line represents the fixed‐effect prediction with shaded areas indicating 95% confidence intervals, and light blue lines show individual wild pig trajectories. (B) Distribution of spatial parameters across six post‐trapping time intervals (days 1, 5, 10, 15, 20 and 30), including distance from trap (km), step length (m), persistence velocity (m day^−1^) and space covered (km^2^). Boxplots represent interquartile ranges with medians shown as bold lines; whiskers extend to 1.5 × IQR, and points indicate outliers.

Across the 30‐day post‐trapping period, we found some evidence that wild pig movement behavior changed substantially over time. The mean distance from the trap ranged from 0.5 to 4.3 km (Fig. 2), with a mean of 1.2 km ± 0.2 SE (Table 2) after trapping events. Our linear mixed‐effect model showed that for each 1‐day increase in time, there was a 0.01 km (±0.07 km, ±95% CI) increase in distance from the trap (GLMM: *P* = 0.011; Table 3; Fig. 3), although this effect was not reflected in comparisons among discrete time points (Kruskal–Wallis: *P* = 0.726; Table 3; Fig. 3). By contrast, we found no strong evidence of temporal change in step length (GLMM _negative binomial_: *P* = 0.957), persistence velocity (GLMM: *P* = 0.873) or space covered (GLMM: *t*_18,77_ = −1.17, *P* = 0.267), and no significant differences across any of the six post‐trapping time points (all Kruskal–Wallis: *P* > 0.60). Spatial overlap remained low and stable (mean = 30.5 ± 1.6% SE; Table 2), with no differences among pairwise time combinations (Kruskal–Wallis: *P* = 0.635). The probability of high overlap (i.e. >30%) remained at <5% throughout the post‐trapping period and showed no significant change over time (GLMM _binomial_: *P* ≥ 0.256).

**Table 2:** Summary of spatial parameters calculated from all 18 wild pigs (*S. scrofa*) collared during trapping events in southeastern Alabama, USA, 2022–2023

Spatial parameter	Mean	95% CI	Min	Max	SD	SE
Distance from the trap (km)	1.3	0.5	0.5	4.3	0.9	0.2
Step length (m)	46.8	7.1	18.7	69.6	14.3	3.4
Persistence velocity (m day^−1^)^†^	2164	436	39	3725	878	207
Space covered (km^2^)	3.6	2.3	<0.01	17.2	4.4	1.1
Overlap area (%)	30.5	12	0	100	22.6	1.6

^†^ Persistence velocity represents speed adjusted for directional consistency (m day^−1^) following.^31^

**Table 3:** Results of GLMMs and Kruskal–Wallis test (K‐W) evaluating short‐term spatial behavior of wild pigs (*S. scrofa*) during the first 30 days post‐trapping in southeastern Alabama, USA, 2022–2023

Spatial parameter		GLMM			K‐W^‡^	
	Estimate (β)^§^	SE	Test statistic	*P*‐value	*χ* ^2^	*P*‐value
Distance from trap	0.01	0.01	*t* = 2.52	0.011	2.83	0.011
Step length	−0.0002	0.004	*Z* = −0.05	0.957	3.58	0.957
Persistence velocity	1.4	9.0	*t* = 0.15	0.873	2.94	0.873
Space covered	−0.05	0.04	*t* = −1.17	0.267	3.20	0.267
Overlap (%)^†^	−0.8 ^c^	4.6	*z* ≤ 0.32	≥ 0.256	11.63	>0.256

^†^ Overlap was modeled separately using logistic regression based on a 30% threshold.

^‡^ Degrees of freedom were 5 for all tests except the logistic regression model (df = 14).

^§^ Estimate refers to the intercept of the logistic regression model.

### 3.2 Social structure effects and long‐term temporal trends

Long‐term temporal variation was assessed in wild pigs tracked from 7 to 138 days (4.5 months) post‐trapping. Over time, spatial parameters generally declined; for each 1‐day increase since capture, step length decreased by 0.2 m (± 0.06 m, ± 95% CI; GLMM: *P* < 0.001; Table 4), persistence velocity decreased by 9.8 m day^−1^ (± 2.7 m day^−1^, ± 95% CI; GLMM: *P* < 0.001), and space covered declined by 0.006 km^2^ (± 0.002 km^2^, ± 95% CI; GLMM: *P* < 0.001). By contrast, distance from the trap did not show a significant temporal trend. Social structure further modulated these patterns. For each 10% increase in the proportion of females in a sounder, step length decreased by 4.9 m (± 0.4 m; ± 95% CI; GLMM: *P* = 0.007; Table 4), and persistence velocity decreased by 238 m day^−1^ (±230 m day^−1^; ± 95% CI; GLMM: *P* = 0.023). Our results suggest that the proportion of females may have a positive effect on distance from the trap (0.2 km increase per 10%; ± 0.5 km, ± 95% CI.; GLMM: *P* = 0.058), whereas no social structure variables significantly influenced (GLMM: all *P* ≥ 0.215) space covered after the trapping event. Body condition did not significantly influence distance from the trap (GLMM: *P* = 0.949; Table 4), step length (GLMM: *P* = 0.123), or space covered (GLMM: *P* = 0.33) following trapping events. However, greater body‐condition index values (reflecting relatively heavier individuals) were associated with slower movements, with persistence velocity decreasing by 67 m day^−1^ (± 66 m/day, ± 95% CI; GLMM: *P* = 0.016) for each 1‐unit increase in the index after the trapping event. Additionally, watershed identity significantly influenced space covered (GLMM: *P* < 0.001).

**Table 4:** Results of GLMMs testing the effects of time since capture (days), social structure and body condition on wild pig (*S. scrofa*) spatial behavior following sounder removal in southeastern Alabama, USA, 2022–2023

Spatial parameter^†^	Predictor^§^	Estimate (β)	SE	*t*‐value	*P*‐value
Distance from trap^‡^	Days since capture	−1.0	1.7	−0.60	0.249
	Sounder size	0.1	0.07	1.43	0.153
	% Female (F)	0.02	0.02	1.01	0.058
	% Adult (A)	0.04	0.02	2.01	0.774
	%F × %A	−0.0006	0.0003	−2.04	0.041
	Body condition^¶^	−0.002	0.04	−0.06	0.949
Step length	Days since capture	−0.2	0.03	−7.21	<0.001
	Sounder size	−0.4	1.0	−0.39	0.695
	% F	−0.5	0.2	−2.66	0.007
	% A	−0.2	0.1	−1.55	0.120
	Body condition	−0.6	0.4	−1.54	0.123
Persistence velocity	Days since capture	−9.8	1.4	−7.20	<0.001
	Sounder size	−90	60	−1.50	0.132
	% F	−23.9	10.5	−2.26	0.023
	% A	−12.9	7.9	−1.62	0.104
	Body condition	−67.1	27.9	−2.40	0.016
Space covered	Days since capture	−0.006	0.001	−4.85	<0.001
	Sounder size	0.07	0.67	0.10	0.922
	% F	−0.13	0.12	−1.09	0.275
	% A	0.01	0.09	0.09	0.928
	Body condition	−0.26	0.27	−0.97	0.334

^†^ All GLMMs were fit a using a Gaussian distribution with individual ID as a random effect and a corAR1 autocorrelation structure.

^‡^ Only the model for distance from the trap retained the interaction between percentage of adults and females; in all other models, the interaction was excluded owing to nonsignificance.

^§^ Sounder size predictor represents the total number of pigs in the sounder, with the collared wild pig not included.

^¶^ A separate model was performed to test the effect of body condition, using a Gaussian distribution and the same covariates as previous model, with body condition included as an additional predictor. Body condition index was calculated as the residuals from a regression of chest girth on body length.

## 4 DISCUSSION

We detected notable variability among wild pigs in their spatial movement patterns after trapping events, which was apparent in their distance travelled from the trap site, step length, rate of movement, and range size. Variability among individuals is common across many wildlife species^46^, ^47^ from carnivores^48^, ^49^ to ungulates^50^, ^51^ and can be influenced by various factors such as age, sex, personality traits, or environmental conditions.^50^, ^52^, ^53^, ^54^, ^55^ Brogi *et al* ^56^ described individual variability in wild boar as reflecting distinct risk‐taking syndromes where some individuals displayed strong site fidelity and human avoidance, and others compensated by selecting open habitats and demonstrating high mobility. These spatial behaviors, referred to as spatial personalities,^47^ highlight how intraspecific differences in movement and habitat use can result from both intrinsic personality traits and environmental interactions, shaping their ability to adapt to exterior stimuli.

Approximately half (55%) of the wild pigs in our study stayed within 1.2 km of the trap site for the entire collaring period: the furthest any wild pigs moved following the trapping event was 6.4 km, and only three animals went further than 2 km from the trap site. Compared to movement patterns reported for wild pigs in other regions, where daily or seasonal space‐use areas often range from ~0.3 to >2 km^2^ depending on habitat, resource availability and social context (e.g. Germany, Italy, Poland, USA, Romania), the distances traveled by isolated pigs in our study were notably small.^57^, ^58^, ^59^, ^60^ Thus, these results suggest that a trapping event that isolates a wild pig from its sounder islikely to not result in that individual leaving the area during the weeks following the event. Other studies with wild pigs have reported similar results. Maillard and Fournier^61^ and Choquenot *et al*.^62^ reported that pigs moved on average 1.8 km in the 2 weeks following the trapping event. Likewise, Sodeikat and Pohlmeyer^63^ documented that only 10% of pigs moved >2 km from the trap site after a drive hunt, with most returning to their central home range within 4–6 weeks. However, when exposed to other types of lethal disturbances, such as dog‐ or drive‐hunting, wild pigs have been reported to travel over much greater distances, often >5 km and sometimes as far as 9 km.^62^, ^63^

Based on expectations drawn from other studies and field observations, we anticipated that wild pigs might exhibit long‐distance movements immediately following the trapping event. However, on the first day following the trapping event, wild pigs in our study travelled distances ranging from 0.4 to 2.1 km. This is consistent with findings from other studies where piglets fled on average 2.2 km to available cover after live‐trapping events.^64^ Variation in flight distance may be influenced by the intensity of the disturbance, prior experiences or pig age.^65^ Moreover, long‐distance travel during the 30‐day window of our study was rare, with our maximum distance traveled in 1 day being 4.5 km. Previous research has reported that wild pigs may travel ≥18 km in a single day to reach resource‐rich areas associated with food, water or other valuable resources,^66^, ^67^, ^68^, ^69^ yet our results suggest that trapping‐related disturbance does not typically elicit short‐term movements of that magnitude.

Although wild pigs in our study progressively increased their mean distance from the trap site during the 30 days following the trapping event, the mean daily increase (0.003 km) that we detected is not biologically significant.^70^ Singer *et al*.^71^ reported different results. They found that wild pigs tend to reduce distance travelled after minor disturbances, such as occasional hunting, with daily distances reduced from 0.6 to 0.2 km after the disturbance. The limited increase in distance from the trap suggests that untrapped pigs may prioritize the benefits of staying within their home range (e.g. access to known resources) over the risks associated with exploring new areas.^72^, ^73^, ^74^

We did not document noticeably heightened activity levels in our wild pigs following the trapping event, which differed from our initial expectations. Instead, we observed relatively consistent movement rates throughout the 30‐day period of interest—below 2 km day^−1^. Although our results suggest a slight decrease in rate of movement over the long‐term—declining by 10 m day^−1^, this reduction does not represent a biologically relevant change in daily movement patterns.^65^ The observed movement rates align with wild pigs in eastern Australia, which moved at speeds of 2.8 km day^−1^,^75^ supporting the idea that after a trapping event, lone wild pigs do not engage in heightened levels of alertness or flight responses. To provide a more detailed comparison, we estimated the hourly movement rates for our wild pigs at ≈0.1 kph. Previous research has shown that speeds <1 kph– are generally associated with routine behaviors—feeding, wallowing, exploring and marking—whereas speeds >2 kph are linked to escaping, excursions and dispersal movements.^11^, ^70^, ^76^ Notably, wild pigs have been reported to move ≤5 kph– during active travel^77^ and can reach speeds of ≤15 kph when alarmed,^78^ though these greater speeds are more reflective of instantaneous speeds rather than sustained daily patterns.

We observed no significant changes in space use for wild pigs after the trapping event, as they continued to cover a similar range size throughout the entire sampling period. Although range size remained consistent, the location of the utilized space appeared to shift. Specifically, pairwise comparisons of daily range across the 30‐day collaring period revealed low spatial overlap, with the probability of exceeding 30% overlap remaining low. This suggests that although pigs used a consistent amount of space, they did not reuse the exact same areas from day to day, indicating spatial reallocation rather than expansion. Our overlap metric captured short‐term space use rather than full home‐range overlap. Because pigs were intensively sampled (96 locations per day), these daily UDs reliably reflected the area used during that day. Because we do not have data on their space use before the trapping event, we could not determine whether the event caused a radical change in their use of space. Nonetheless, our study animals remained relatively consistent in the range size that they used after the trapping event. Over the 30‐day period following the trapping events our wild pigs showed a mean space covered of 3.2 km^2^. This range size estimate is generally less than what has been reported in previous studies where wild pigs did not experience any disturbance.^57^, ^65^, ^70^ Similar range sizes to ours have been reported in Georgia, USA (3.1 km^2^), where that population had been exposed to both hunting and trapping.^39^ However, our range size estimates were considerably larger than other studies that also experienced a control event—between 0.3 and 1.5 km^2^.^79^, ^80^, ^81^, ^82^, ^83^ In Alabama, previous research showed that wild pigs under high hunting pressures during a collaring period of 1.4 to 3.5 months had an average range size of 2.7 km^2^, whereas those experiencing lower hunting pressure had range sizes nearly twice as large (4 km^2^).^84^ Range sizes can be influenced by various abiotic factors such as latitude, vegetation, food availability and water sources,^57^ which may explain some of the variation observed between our study and others.

We detected a positive association between the percentage of females in the sounder and distance from the trap during the entire collaring period. Wild pigs are known to form female‐led groups with strong social cohesion,^85^ and females often influence movement patterns—possibly as a consequence of their role in seeking safer or resource‐rich areas for their sounder.^17^ Similar patterns have been observed in other socially structured species, such as elephants (*Loxodonta africana*) and giraffes (*Giraffa camelopardalis tippelskirchi*), where matriarchs often lead group movement, especially during disturbance.^86^, ^87^ Based on these dynamics, we anticipated that wild pigs from female‐dominated sounders might remain closer to the trap site, maintaining spatial cohesion. However, our results showed the opposite: individuals from more female‐dominated sounders tended to move farther from the trap. Given the limited data on post‐trapping spatial responses in female wild pigs, any firm conclusion regarding the cause of this pattern would be speculative. It remains possible that the influence of female leadership within the sounder could condition movement dynamics, but more research is needed to determine the underlying factors driving these behaviors.

We found a negative association between body condition and activity levels following the trapping event. Specifically, we observed that pigs in better condition exhibited slower velocities, with a decrease of 67 m per day following the trapping event. Likewise, movement behavior in other species has been shown to vary based on resource availability and landscape configuration, which directly influence body condition. For example, Doherty *et al*.^88^ demonstrated that greater resource availability in linear remnants reduced activity area size and movement rates in eastern bearded dragons (*Pogona barbata*). In another study, moose (*Alces alces*) in poorer condition exhibited greater activity levels during stressful conditions compared to those in better condition,^89^ although the animals in that study were not exposed to a stressful event such as trapping. Previous studies on wild pigs also have shown that spatial behavior and activity are primarily driven by seasonal changes in food availability, with individuals accustomed to reliable food sources tending to exhibit less activity.^90^ These patterns also may intersect with fear ecology, whereby perceived risk from human presence or disturbance influences space usage and activity levels. Invasive species such as wild pigs may respond to such perceived risks in complex, context‐dependent ways in their introduced ranges. This link between body condition, perceived risk and post‐trapping behavior warrants further investigation, particularly given that removal programs are most effective when integrating multiple control approaches concurrently.

## 5 CONCLUSION

Our data suggest that the likelihood of an infected pig, isolated from its original sounder, dispersing beyond the trapping area to infect animals elsewhere appears to be low. Despite some individual differences, most wild pigs did not travel far from the trap site during the 30‐day period following a trapping event. Their movement rates remained slow and spatial metrics consistent, indicating minimal disruption to spatial parameters. This stability reduces the likelihood of disease spread at a population level under trapping conditions. However, spatial behavior can vary depending on latitude,^58^ behavioral context and sounder structure. For instance, in areas where wild pigs exhibit territorial behavior,^28^, ^39^, ^40^ disease transmission risk is less than in regions where groups overlap extensively.^12^, ^15^, ^91^ Lone wild pigs present a unique risk, as they may join new sounders even while remaining within the spatial limits of their original sounder. In our study, this occurred in one instance, confirmed by camera‐trap images.

Undoubtedly, control efforts designed to eliminate wild pig populations in the case of a novel disease outbreak is likely to incorporate whole sounder removal,^12^ or other trapping strategies^10^ to maximize probability of success. However, it is highly likely that not every sounder will be trapped and euthanized in a single trapping effort. Our data indicate that management programs such as these will not be negatively impacted by such occurrences. Rather, our findings indicate that these efforts may be more effective than previously anticipated.^92^ Wild pigs that remain isolated after trapping are likely to stay near the trap site, enabling managers to target them with continued trapping efforts. Our findings indicate that these occurrences will not overly hinder the success of management programs. As mentioned before, wild pigs remained ≤1.2 km of the trap site—a distance well within the effective range of modern trapping systems. Considering that traps can influence wild pig movements over an area of 8.87 km^2^,^93^ pigs left behind are likely to remain within range of the initial trap or nearby traps, increasing the likelihood of recaptures through sustained efforts.

It should be noted that our data are limited to females that are currently in a sounder. Space use of males that are associated with a sounder, yet are not trapped when the sounder is captured, remains an important area for further study. Male wild pigs are more likely to disperse widely,^9^, ^94^ interact with multiple sounders,^56^ and increase the risk of disease transmission over larger areas,^95^, ^96^ particularly during active breeding periods.^97^ By contrast, female‐led sounders exhibit high site fidelity^98^ and limited dispersal, posing a lower immediate risk of spreading disease. However, the strong cohesion of sounders may require expanded control zones for systematic removal. Adapting trapping strategies to account for these behavioral differences, based on the structure and condition of the population, improves management effectiveness.

## AUTHOR CONTRIBUTIONS

SSD designed the study. SGM and MM collected the data. SGM and JV analyzed the data. All authors drafted the manuscript to its final version.

## FUNDING INFORMATION

This research was funded by the Alabama Soil and Water Conservation Commitee through the USDA Natural Resources Conservation Service's Feral Swine Eradication and Control Pilot Program.

## CONFLICT OF INTEREST

Authors declare no conflict or competing interests.

## Supporting information


**Data S1.** Supporting Information.

## Data Availability

The data that support the findings of this study are available from the corresponding author upon reasonable request.
