# Supplementary material for: Spatial behavior of socially isolated wild pigs ( Sus scrofa) following sounder removal via trapping
Source: Pest Manag Sci. 2026 Mar 12;82(6):5225–36. doi: 10.1002/ps.70630 (PMC13158450; doi:10.1002/ps.70630)
Supplement: Supplementary file 1 — Data S1. Supporting Information. [file PS-82-5225-s001.docx]

Supplemental Material for

Spatial behavior of socially isolated wild pigs (*Sus scrofa*) following sounder removal via trapping

Sebastian Gomez-Maldonado^1*^, Matthew T. McDonough ^1^, Jonathon Valente ^1,2^, D. Mark D. Smith^1^, & Stephen S. Ditchkoff ^1^.

^1^College of Forestry, Wildlife and Environment, Auburn University, Auburn, AL 36849

^2^U.S. Geological Survey, Alabama Cooperative Fish & Wildlife Research Unit, Auburn, AL 36849

*Corresponding author: Sebastian Gomez-Maldonado

Email: szg0159@auburn.edu

This PDF file includes:

- **Supplemental Material 1** – Model selection of movement models (p. 2-3).
- **Supplemental Material 2** – Statistical distribution values for model selection (p. 4-6).
  - SM2 Table 1 – Values for social structure analyses (p. 4-5).
  - SM2 Table 2 – Values for body condition analyses (p. 6).
- **Supplemental Material 3** –Pearson’s correlation matrix for variable selection (p. 7).
- **Supplemental Material 4** – Model comparison of generalized linear mixed-effect models (GLMMs) with temporal autocorrelation corrections for social structure and body condition models (p. 8)
- **Supplemental Material 5** - Visual representations of temporal autocorrelation correction in all generalized linear mixed-effect models (GLMMs) (p. 9-11)

Any use of trade, firm, or product names is for descriptive purposes only and does not imply endorsement by the U.S. Government.

**Supplemental Material 1.** Model selection of movements models for each individual wild pig (*Sus scrofa*) to calculate area covered. The best model was selected using the delta Akaike’s information criterion (AIC) obtained after fitting movement models to location data from all pigs, the lowest value represents the best model. Prior to fitting hypothetical movement models, prototype models with guesstimated parameters (*i.e.,* Brownian motion with a particular diffusion rate) were calculated from location data. Model selection involved two phases: (1) fitting simpler models to improve numerical converge and (2) adjusting the autocorrelation and deterministic trend models.

|  |  |  |  | ∆AICc |  |  |  |
| --- | --- | --- | --- | --- | --- | --- | --- |
| Pig ID | ^†^ IID | IID  anisotropic | ^‡^ OU | OU anisotropic | ^§^ OUF | OUF anisotropic | ^¶^ OUf anisotropic |
| PIG 1 |  | 241.37 | 216.25 | 0.00 | 217.91 | 1.97 | 60.48 |
| PIG 2 |  |  |  | 54.99 | 483.89 | 0.00 | 1770.62 |
| PIG 3 |  |  |  | 36.23 | 328.23 | 0.00 | 4568.88 |
| PIG 4 |  |  |  | 1683.57 | 513.62 | 0.00 | 4792.07 |
| PIG 5 |  |  |  | 913.06 | 616.77 | 0.00 | 4644.70 |
| PIG 6 | 1183.65 | 0.00 |  |  |  |  |  |
| PIG 7 |  |  |  | 1981.28 | 186.81 | 0.00 | 5432.62 |
| PIG 8 |  |  |  | 1535.83 | 86.36 | 0.00 | 5827.34 |
| PIG 9 |  |  |  | 1119.58 | 89.03 | 0.00 | 703.92 |
| PIG 10 |  |  |  | 211.85 | 22.10 | 0.00 | 396.90 |
| PIG 11 |  |  |  | 176.92 | 79.43 | 0.00 | 373.04 |
| PIG 12 |  |  |  | 3914.84 | 1358.86 | 0.00 | 5249.11 |
| PIG 13 |  |  |  | 960.69 | 858.94 | 0.00 | 3241.22 |
| PIG 14 |  |  |  | 737.21 | 684.82 | 0.00 | 5258.38 |
| PIG 15 |  |  |  | 102.52 | 28.10 | 0.00 | 366.82 |
| PIG 16 |  |  |  | 361.82 | 289.12 | 0.00 | 2176.19 |
| PIG 17 |  |  |  | 361.82 | 289.12 | 0.00 | 2176.19 |
| PIG 18 |  |  |  | 27.09 | 50.39 | 0.00 | 237.19 |

^†^ “IDD” denotes the uncorrelated bi-variate Gaussian model and stands for Independent and Identically Distributed.

^‡^ ”OU” denotes the continuous-position Ornstein-Uhlenbeck model.

^§^ “OUF” denotes the continuous-velocity Ornstein-Uhlenbeck-F model and stands for Ornstein-Uhlenbeck Foraging.

^¶^ “OUf” denotes the OUF model where the two autocorrelation timescales cannot be statistically distinguished.

**Supplemental Material 2**. Chi-square (χ^2^) values for model fit selection of spatial parameters for all generalized linear mixed-effect models (GLMMs). Each model was evaluated across three statistical distributions. The visual representation of the selected model is included. The red line represents the chi-square value of the simulated dataset compared to the actual distribution of the original data. A model was considered a reasonable fit if the chi-square line fell within the upper or lower 0.025 quantiles of the original distribution. Selected model is indicated in bold.

**SM2 Table 1.** Statistical distribution values for selection process for short-term temporal variation analyses.

| Spatial parameter | Normal (χ^2^) | Poisson (χ^2^) | Negative binomial (χ^2^) | Visual representation |
| --- | --- | --- | --- | --- |
| Distance from trap | **0.9** | 1 | 1 | 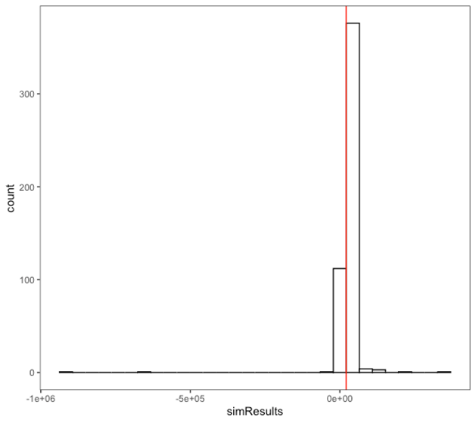 |
| Step length | 0 | 0 | **0.278** | 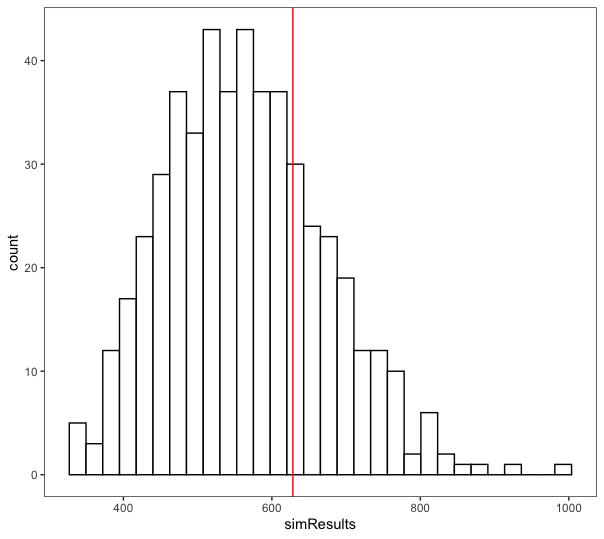 |
| Persistent velocity | **0.818** | 0 | 0.77 | 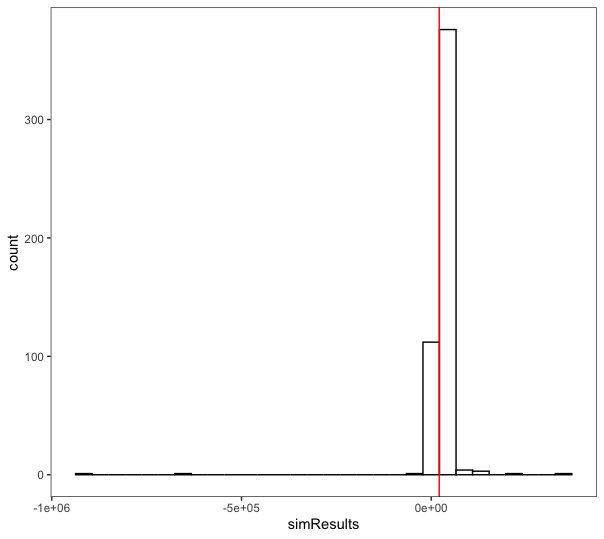 |
| Space covered | **0.716** | 0 | 0.186 | 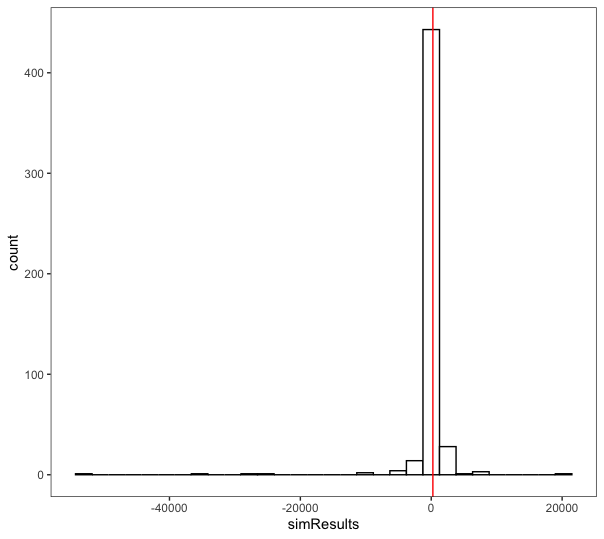 |

**SM2 Table 2.** Statistical distribution values for selection process of generalized linear mixed-effect models (GLMMs) for social structure and body condition analyses.

| Spatial parameter | Normal (χ^2^) | Poisson (χ^2^) | Negative binomial (χ^2^) | Visual representation |
| --- | --- | --- | --- | --- |
| Distance from trap | **0.65** | 1 | 1 | 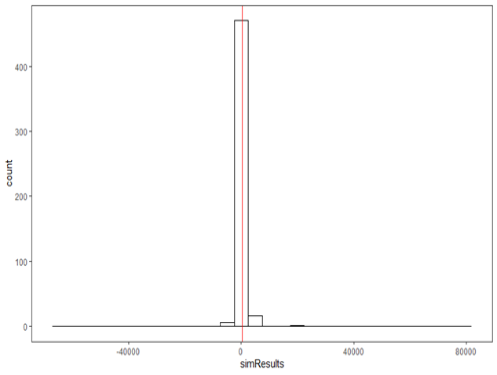 |
| Step length | **0.932** | 0 | 0.114 | 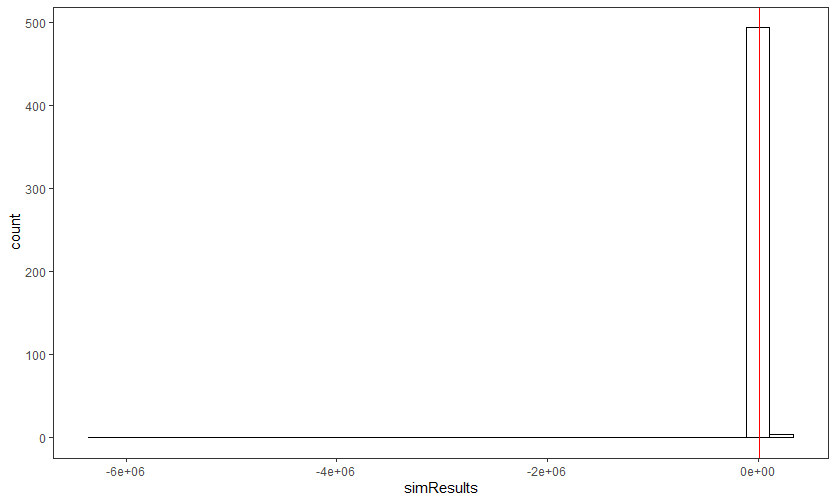 |
| Persistent velocity | **0.866** | 0 | 0.618 | 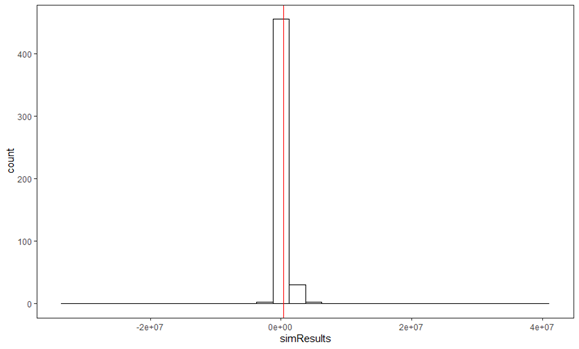 |
| Space covered | **0.298** | 1 | 1 | 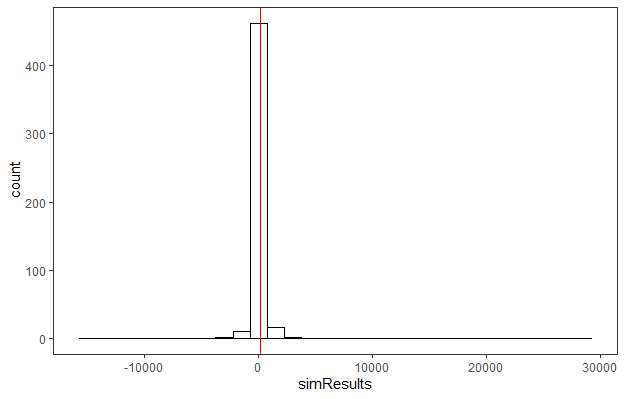 |

**Supplemental Material 3.** Pearson’s Correlation matrix. A variable autocorrelation analysis was performed to select variables that were used in the generalized linear mixed-effect models (GLMMs). Variables with a coefficient > ± 0.7 were not included in the same model to avoid multicollinearity issues. Circle size represents the magnitude of the correlation index, for both positive and negative values. Blue scale represents the positive correlation index, while red represents the negative correlation index.


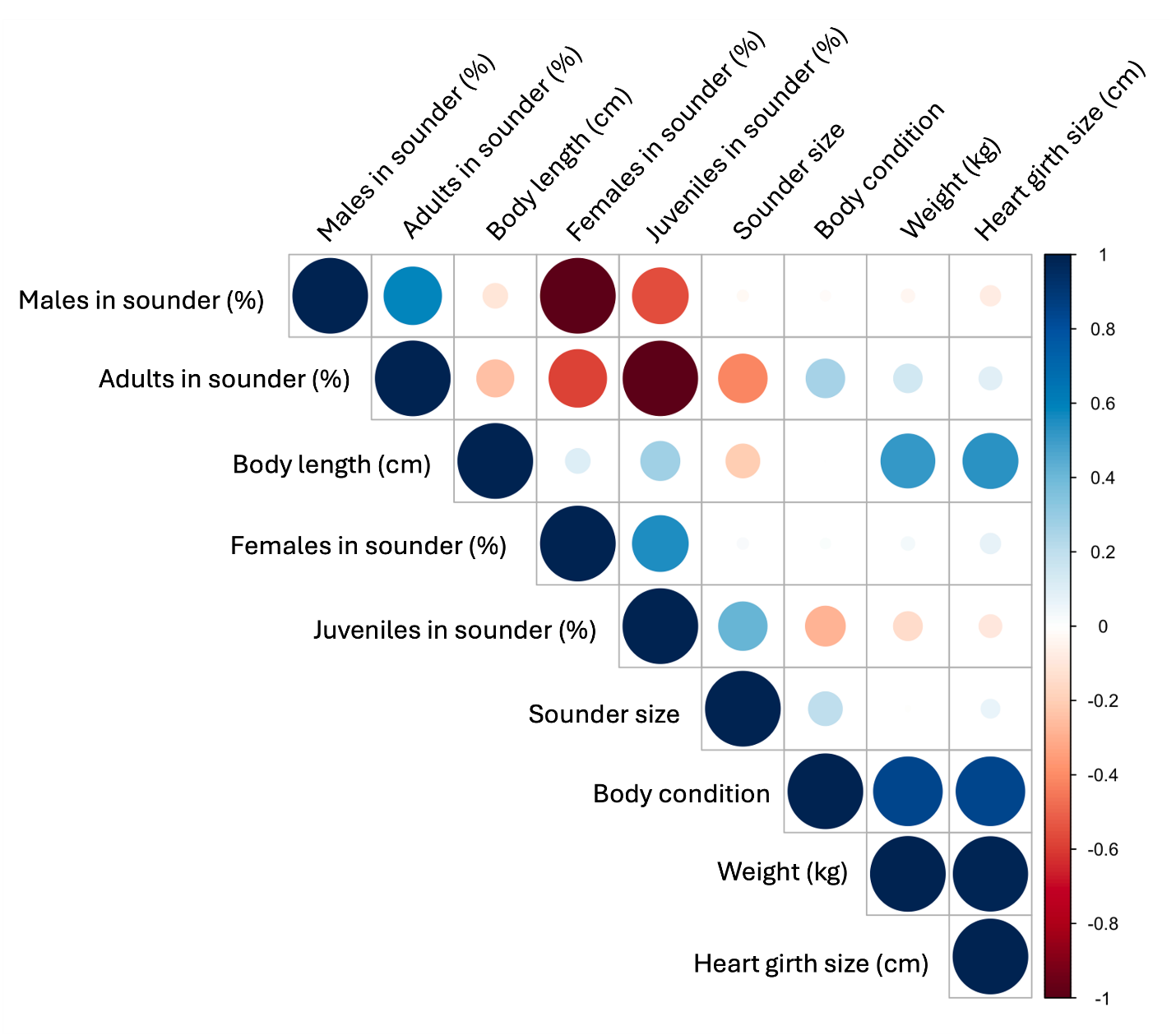


**Supplemental Material 4:** Model comparison of improvement with and without consideration of temporal autocorrelation in generalized linear mixed-effect models (GLMMs). Smaller AIC values indicate an improvement in model performance. Two sets of GLMMs were performed, one to test the effect of social structure and another to test the effect with body condition.

| Models | Spatial parameter | Temporal autocorrelation | Intercept (ẞ0) | SE | df | AICc | ∆AIC |
| --- | --- | --- | --- | --- | --- | --- | --- |
| Social Structure | Distance to trap (km) | Fixed | -1.029 | 1.716 | 11 | 1461.1 | 0 |
|  |  | Not fixed | -0.910 | 1.764 | 10 | 2207.0 | 745.91 |
|  | Step Length (m) | Fixed | 94.96 | 19.336 | 10 | 9683.3 | 0 |
|  |  | Not fixed | 97.18 | 19.063 | 9 | 9829.8 | 146.48 |
|  | Velocity (m/day) | Fixed | 4964 | 1111.227 | 10 | 17982.2 | 0 |
|  |  | Not fixed | 4978 | 1096.880 | 9 | 18063.4 | 81.14 |
|  | Area covered (km^2^) | Fixed | 11.91 | 12.359 | 10 | 3026.6 | 0 |
|  |  | Not fixed | 11.95 | 12.378 | 9 | 3144.6 | 118.05 |
| Body Condition Models | Distance to trap (km) | Fixed | 1.730 | 1.738 | 11 | 1370.3 | 0 |
|  |  | Not fixed | 1.941 | 1.729 | 10 | 2032.6 | 662.28 |
|  | Step Length (m) | Fixed | 72.42 | 17.917 | 11 | 8446.1 | 0 |
|  |  | Not fixed | 77.16 | 17.321 | 10 | 8586.3 | 140.17 |
|  | Velocity (m/day) | Fixed | 2984 | 1201.96 | 11 | 15682.5 | 0 |
|  |  | Not fixed | 2922 | 1237.20 | 10 | 15760.3 | 77.88 |
|  | Area covered (km^2^) | Fixed | 8436 | 11.780 | 11 | 2695.1 | 0 |
|  |  | Not fixed | 8.511 | 11.776 | 10 | 2796.3 | 101.23 |

**Supplemental Material 5.**Autocorrelation function (acf) plots showing temporal autocorrelation in residuals from each generalize linear mixed-effect models (GLMMs). The x-axis (lag) represents the number of 15-minute time steps between observations. Blue dotted lines denote 95% confidence limits for autocorrelation estimates. . Two sets of GLMMs were performed, one to test the effect of social structure and another to test the effect with body condition for all spatial parameters.

|  |  | Temporal autocorrelation scatterplots | |
| --- | --- | --- | --- |
|  |  | Not fixed | Fixed |
| Social Structure Models | Distance to trap | 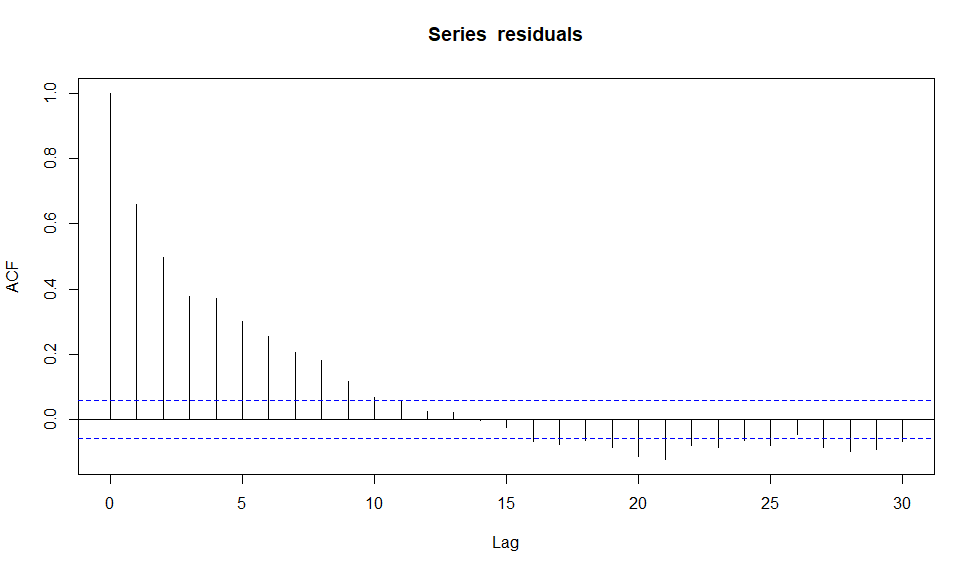 | 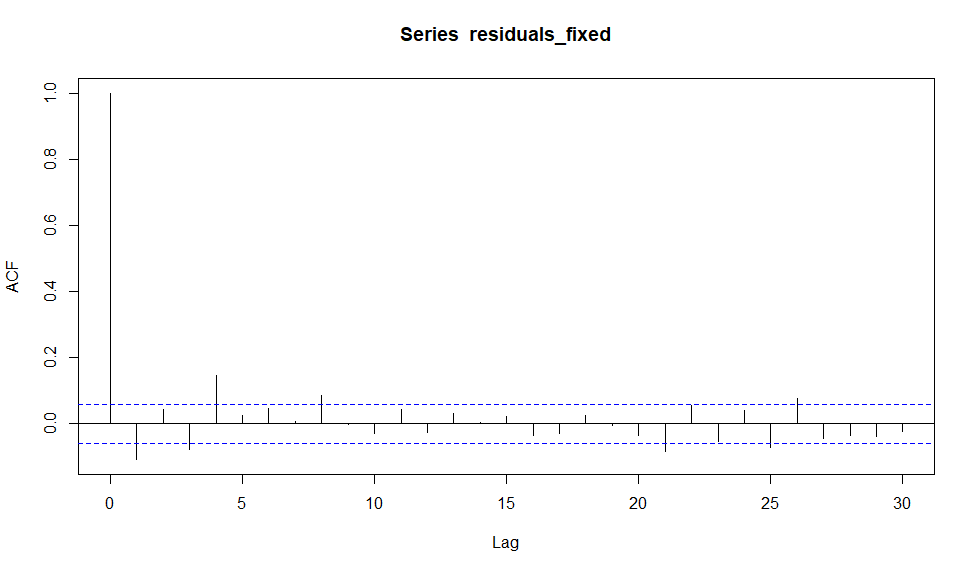 |
|  | Step Length | 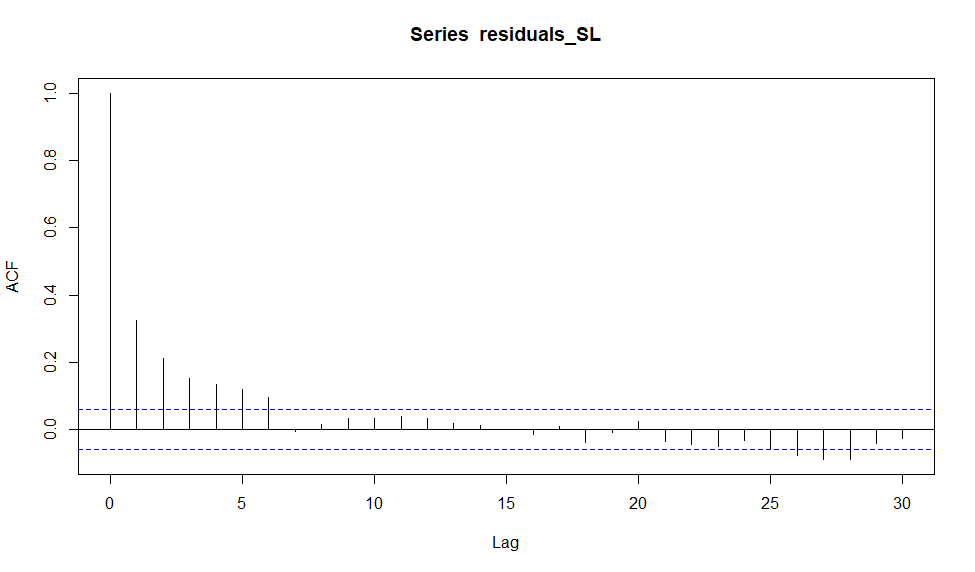 | 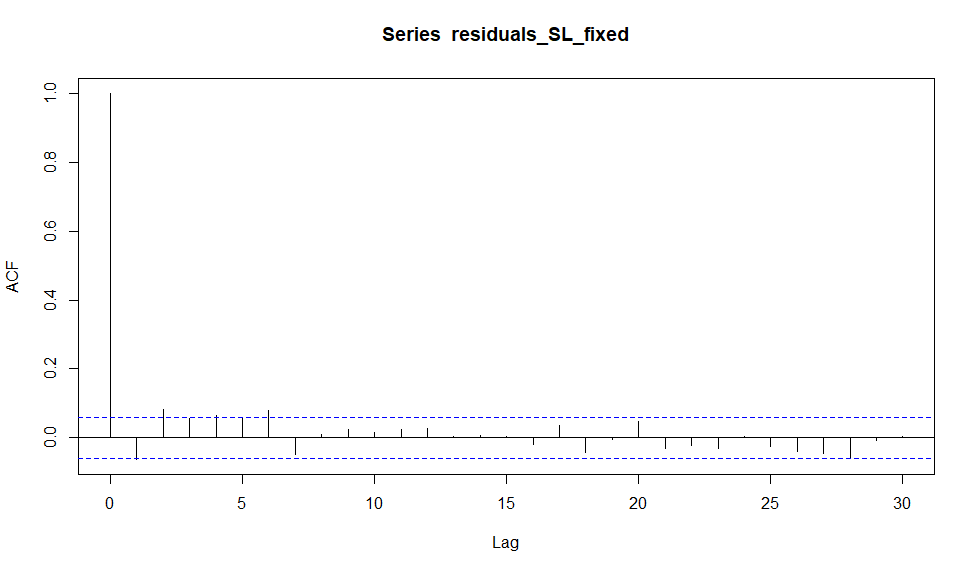 |
|  | Velocity | 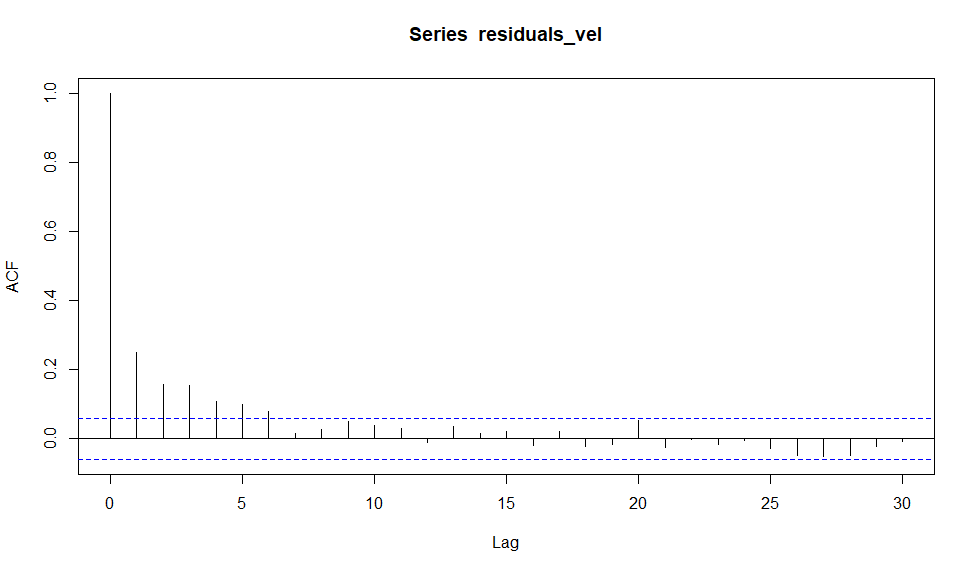 | 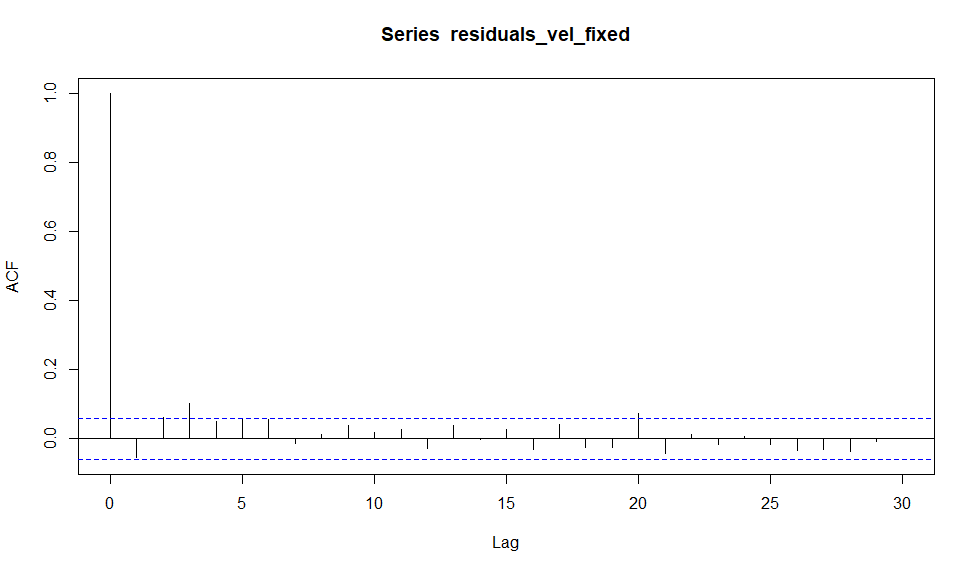 |
|  | Area covered | 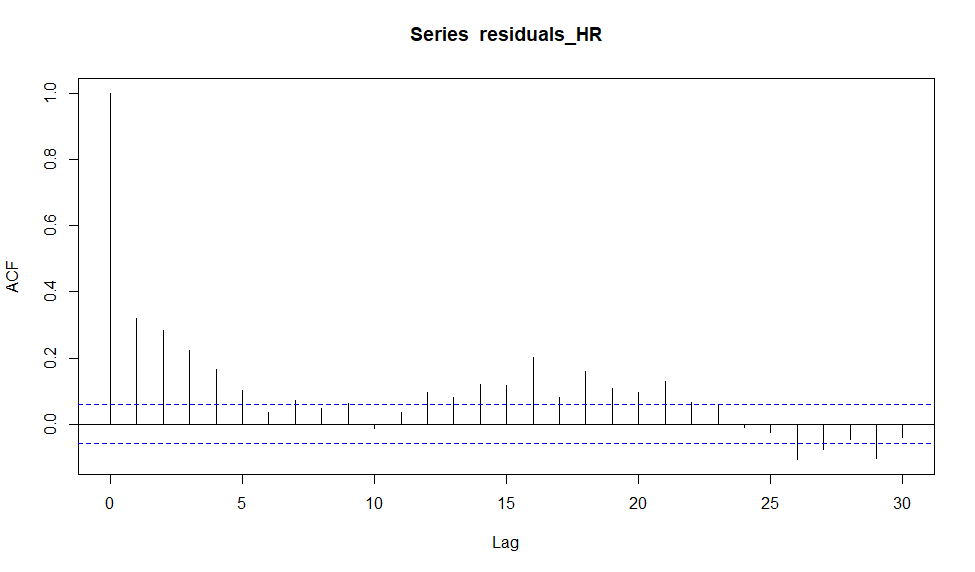 | 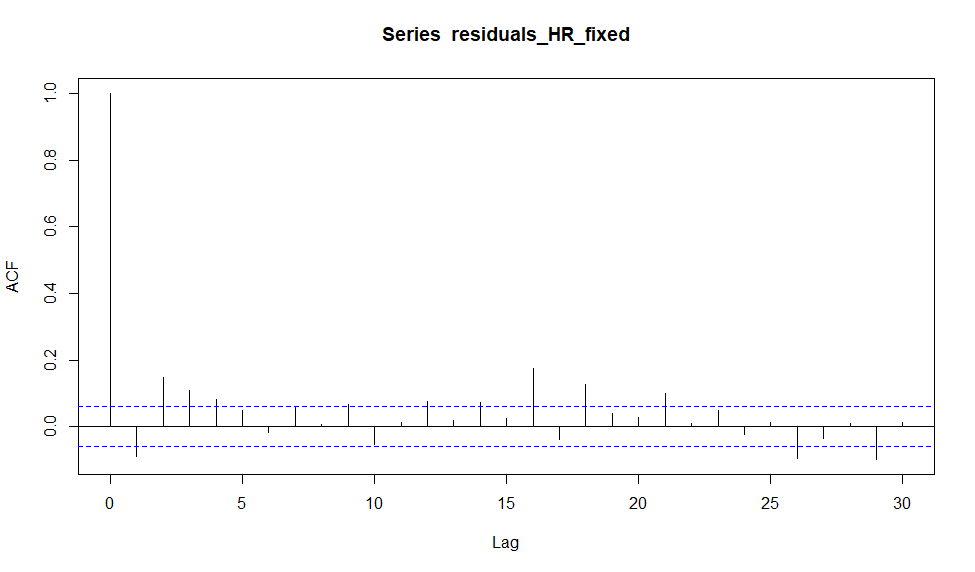 |
| Body Condition Models | Distance to trap | 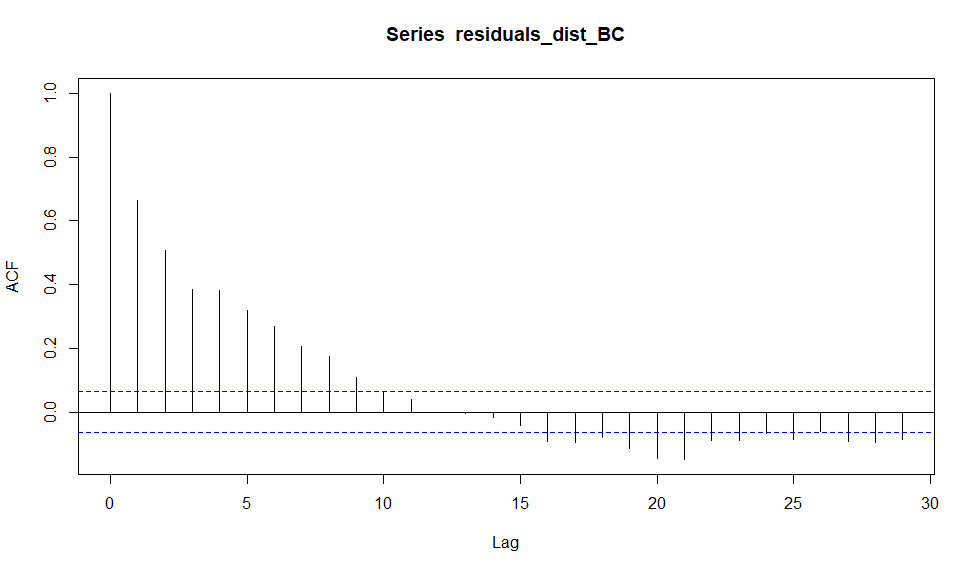 | 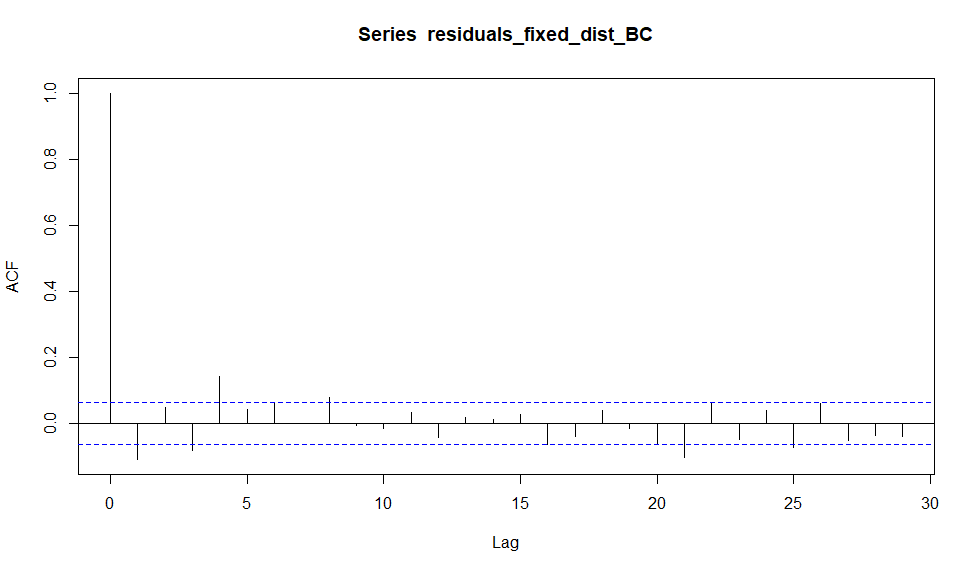 |
|  | Step Length | 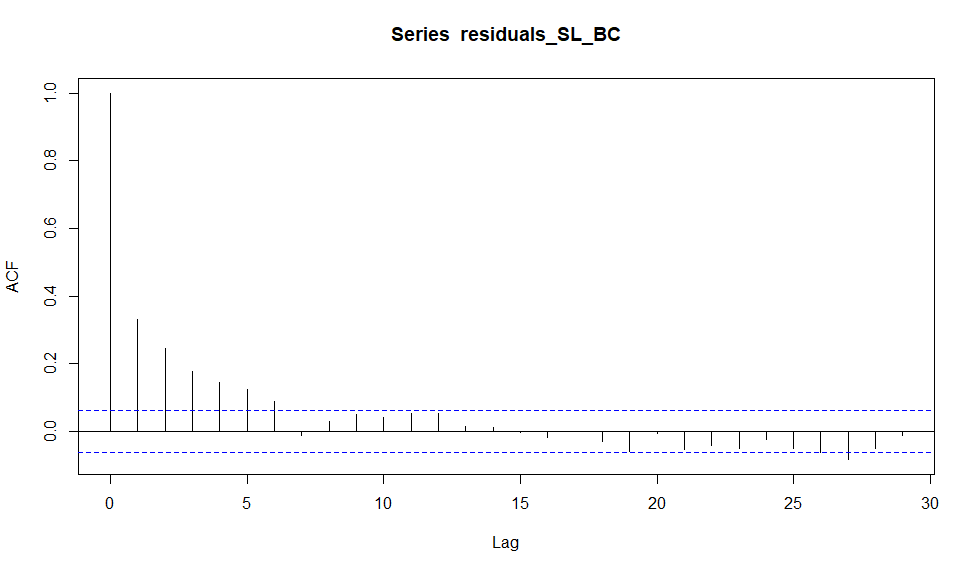 | 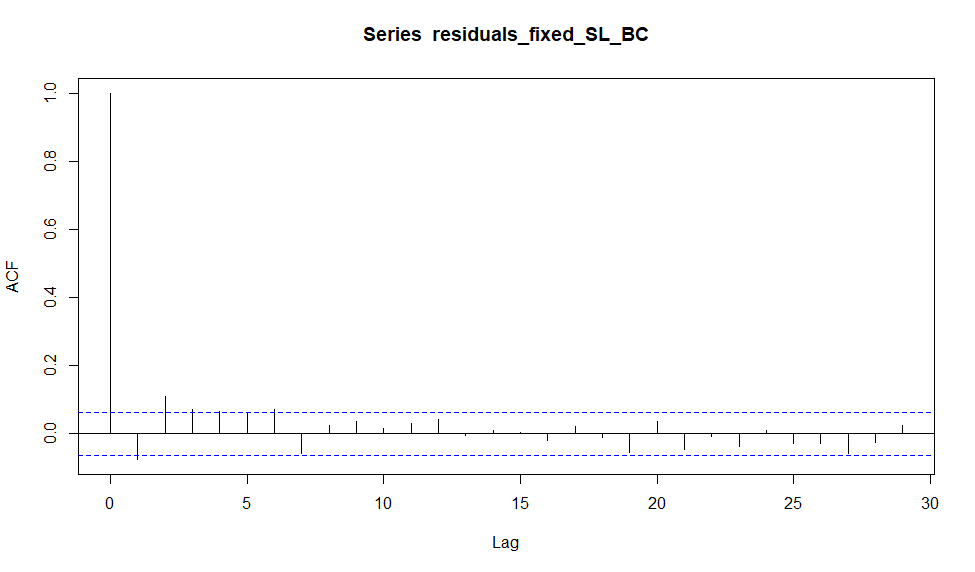 |
|  | Velocity | 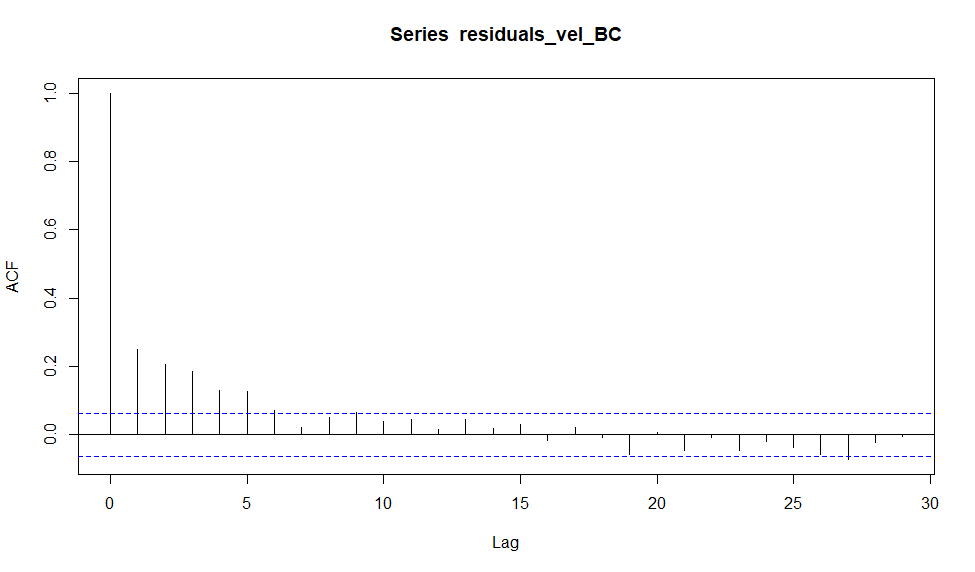 | 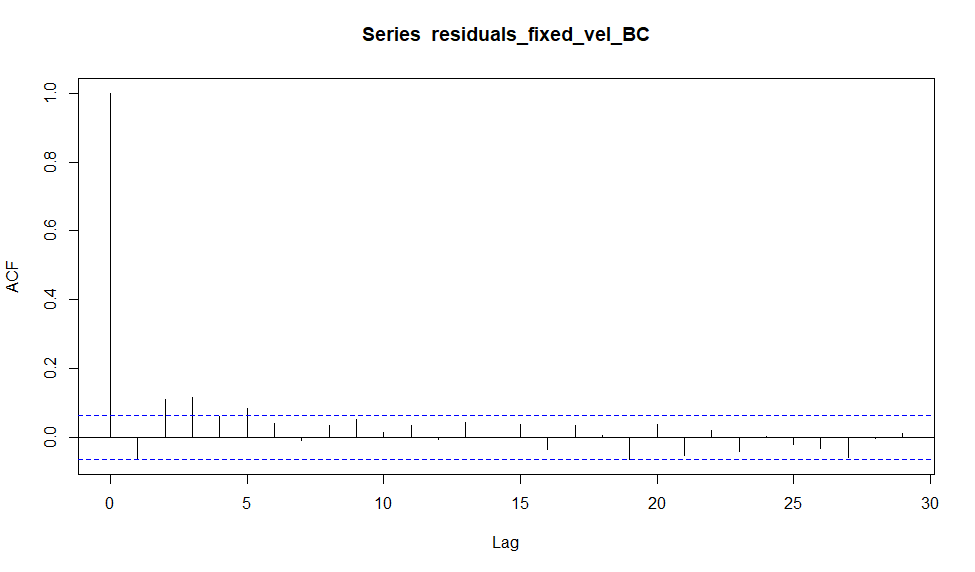 |
|  | Area covered | 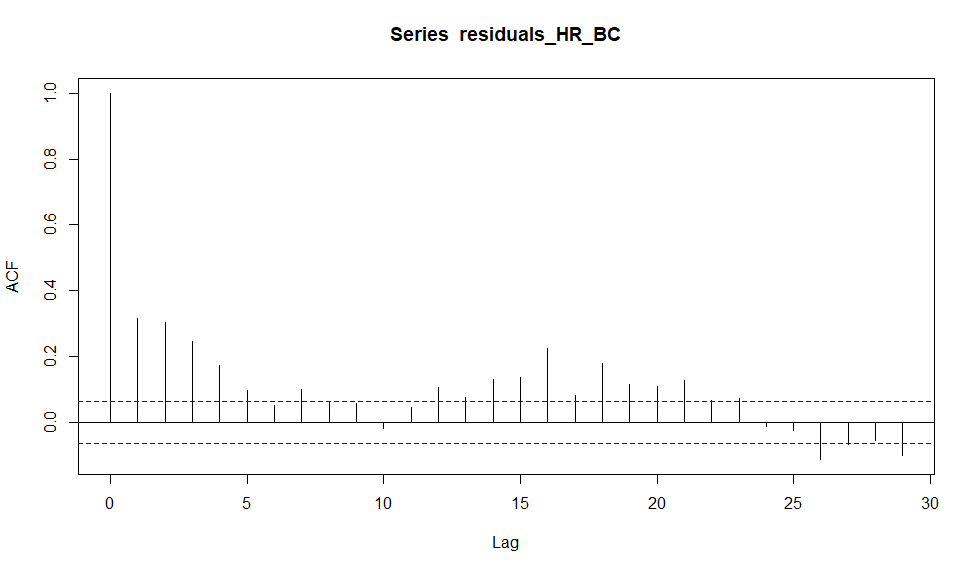 | 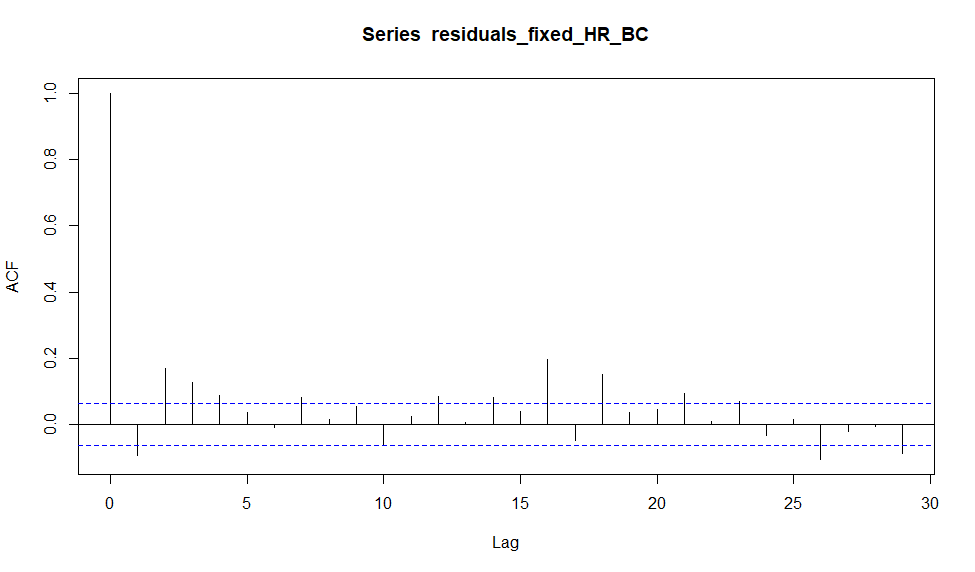 |
